# Relative suppression of pyroptosis- and necroptosis-related transcriptional programs and enrichment of ferroptosis-related signatures are associated with fatal sepsis: multi-cohort bulk and single-cell transcriptomic evidence

**DOI:** 10.3389/fcell.2026.1885384

**Published:** 2026-09-25

**Authors:** Jing Xu, Peng Wang, Mingzhao Xu

**Affiliations:** 1 Department of Tuberculosis, Public Health Clinical Center Affiliated to Shandong University, Jinan, China; 2 Infectious Diseases Unit, Tianjin 4th Center Hospital, Tianjin, China

**Keywords:** DAMP, necroptosis, pneumonia, programmed cell death, pulmonary infection, pyroptosis, sepsis, single-cell RNA sequencing

## Abstract

**Background:**

Sepsis is a life-threatening syndrome caused by a dysregulated host response to infection and remains a major cause of mortality in critically ill patients. Multiple forms of programmed cell death (PCD) may contribute to immune dysregulation and organ injury, but their transcriptional patterns across disease severity and outcome remain incompletely defined.

**Methods:**

We performed an integrative transcriptomic analysis of five public sepsis cohorts, including single-cell RNA sequencing datasets GSE167363 and GSE151263 and three bulk transcriptomic cohorts. Primary outcome analyses focused on cohort-defined fatal sepsis (28-day non-survivors in GSE65682 and the deposited sepsis-death group in GSE63042), whereas pneumonia/ARDS comparisons were treated as secondary exploratory analyses. PCD-related transcriptional programs were evaluated across clinical groups and immune-cell populations. Six candidate genes were further assessed by RT-qPCR in LPS-stimulated RAW264.7 and THP-1 cells as targeted *in vitro* transcriptional validation.

**Results:**

In GSE65682, 28-day non-survivors showed lower pyroptosis, necroptosis and apoptosis program scores but higher ferroptosis scores than survivors, and higher pyroptosis scores were associated with better 28-day survival. GSE154918 instead showed broader increases in PCD-related program scores from healthy controls to infection/sepsis groups, reflecting a disease-versus-control contrast rather than an outcome contrast. In the LPS model, CASP1, RIPK3 and NLRP3 were upregulated, whereas SIRT1, BCL2 and HIF1A were downregulated. These cell experiments assessed acute inflammatory transcriptional responsiveness and were not intended to reproduce the mortality-associated direction observed in patient cohorts.

**Conclusion:**

Multi-cohort analyses identified outcome-associated PCD transcriptional patterns in sepsis, including relative suppression of pyroptosis- and necroptosis-related programs and enrichment of ferroptosis-related signatures in non-survivors. The LPS experiments provide complementary mRNA-level evidence that selected PCD genes are transcriptionally responsive to inflammatory stimulation, but they do not constitute directional or functional validation of the fatal-sepsis signature. Functional and prospective clinical validation remains required.

## Introduction

Sepsis, now recognized as a leading cause of mortality in intensive care units (ICUs) worldwide, results from a dysregulated host response to infection that leads to widespread tissue damage and multi-organ dysfunction. Globally, sepsis accounts for over 11 million deaths annually, representing approximately 20% of all-cause mortality. Despite decades of research and advances in antimicrobial therapy and hemodynamic support, targeted interventions remain insufficient, largely because the molecular underpinnings of sepsis are extraordinarily complex, involving multiple overlapping and interactive forms of programmed cell death (PCD) (Singer et al., 2016; Rudd et al., 2020; van der Poll et al., 2021). In the present study, “fatal sepsis” is used operationally to denote the outcome-defined death/non-survivor groups available in the source cohorts: 28-day non-survivors in GSE65682 and the deposited sepsis-death group in GSE63042; it is not treated as a separate diagnostic entity.

Recent studies have expanded our understanding of how regulated cell death is associated with sepsis outcomes. Beyond classical apoptosis, non-apoptotic forms including necroptosis, pyroptosis, and ferroptosis have been implicated in immune dysfunction and tissue injury. These pathways can be accompanied by release of damage-associated molecular patterns (DAMPs), including HMGB1, mitochondrial DNA, and ATP, which may amplify inflammatory signaling through pattern-recognition receptors (Qu et al., 2022; Linkermann et al., 2014; Kang et al., 2018). Importantly, transcript abundance alone does not establish execution of these cell-death pathways, which also depends on post-transcriptional and post-translational regulation.

Important gaps remain regarding how PCD-related transcriptional programs vary by immune-cell lineage, disease phase, and clinical outcome in sepsis. NLRP3/caspase-1 signaling is linked to pyroptosis, whereas RIPK1/RIPK3/MLKL signaling is central to necroptosis; however, functional pathway execution requires biochemical evidence beyond gene-expression measurements (He et al., 2016; Pasparakis and Vandenabeele, 2015; Galluzzi et al., 2018). Integrating bulk and single-cell transcriptomic data can therefore identify reproducible associations and candidate regulatory relationships, but cannot by itself establish causality.

Accordingly, the primary objective of this study was to determine whether PCD-related transcriptional programs differ between survivors and non-survivors and whether the outcome-associated patterns are reproducible across independent bulk transcriptomic cohorts. Single-cell RNA sequencing was used to localize these transcriptional programs to immune-cell populations, and targeted RT-qPCR was used to test the inflammatory transcriptional responsiveness of selected candidate genes. Pneumonia-associated sepsis and ARDS analyses were retained as secondary exploratory analyses because pulmonary infection is a common precipitating source of sepsis (Hotchkiss et al., 2016; Delano and Ward, 2016), but they were not used as the principal evidence for the fatal-outcome conclusion.

Analytical workflow: the primary outcome analysis used the GSE65682 28-day non-survivor-versus-survivor comparison together with the GSE63042 sepsis-death-versus-survivor comparison. Additional disease-versus-control analyses were used to distinguish general sepsis-associated transcriptional activation from outcome-associated changes. ScRNA-seq cohorts were used to resolve cell-type-specific PCD patterns and patient-level pseudobulk effects; cross-platform comparisons assessed consistency; and selected candidate genes were evaluated by RT-qPCR in LPS-stimulated RAW264.7 and THP-1 cells. Pneumonia/ARDS analyses were considered exploratory contextual analyses. The workflow was designed to identify transcriptional associations rather than establish causal pathway execution.

## Methods

### Data sources and cohort verification

This study reanalyzed five public transcriptomic cohorts obtained from the NCBI Gene Expression Omnibus (GEO). Single-cell RNA sequencing (scRNA-seq) of peripheral blood mononuclear cells (PBMCs) came from GSE167363 (GPL24676, Illumina NovaSeq 6000; 12 libraries from seven subjects: two healthy controls and five patients with Gram-negative sepsis sampled at sepsis recognition [T0] and 6 h later [T6], including three survivors and two non-survivors; PMID 34558746) and GSE151263 (GPL20301, Illumina HiSeq 4000; seven frozen PBMC libraries from seven patients with pneumonia-associated sepsis, 4 with sepsis only and 3 with sepsis plus acute respiratory distress syndrome [ARDS]; PMID 32554932). Bulk transcriptomes comprised GSE63042 (GPL9115; RNA-seq RPM; 129 whole-blood samples: 23 systemic inflammatory response syndrome [SIRS], 24 uncomplicated sepsis, 21 severe sepsis, 33 septic shock and 28 sepsis deaths), GSE65682 (GPL13667, Affymetrix HG-U219; 801 whole-blood samples including 479 sepsis patients with 28-day outcomes [365 survivors, 114 non-survivors], 108 community-acquired pneumonia [CAP], 84 hospital-acquired pneumonia [HAP] and 32 non-infectious critically ill controls) and GSE154918 (GPL20301; 105 whole-blood RNA-seq samples: 40 healthy, 12 uncomplicated infection, 20 sepsis, 19 septic shock and 14 within-patient follow-up samples excluded from cross-sectional comparisons). Every accession, platform and sample-level group was verified against the official GEO Series, Sample and SOFT records; sample-level inclusion is documented in Metadata/dataset_manifest.csv.

### Bulk transcriptome processing and scale audit

GSE63042 RPM values (linear scale) were transformed once to log2 (RPM+1). GSE154918 supplied log2-normalized counts and GSE65682 supplied RMA-processed values already in log2 space; both were used without re-transformation after a scale audit (min/max 1.18/13.54 for GSE65682; 0.00/23.90 for GSE154918). GSE63042 transcript identifiers were mapped to gene symbols via the deposited gene_name annotation; GSE65682 probes were mapped to gene symbols with Bioconductor hgu219. db (24,550 probes mapped, 11,537 unique genes), averaging probes per gene.

### Differential expression

Two-group differential expression was assessed with Welch’s t-test on the log2 scale; the effect direction is always reported as case minus control. Benjamini–Hochberg adjustment was applied across all tested genes, with significance defined as adjusted P < 0.05 and |log2 fold change| ≥ 0.5. Four bulk contrasts were used for the main cross-cohort DEG comparison in Figure 1D: (1) GSE63042 all sepsis groups (n = 106) versus SIRS (n = 23); (2) GSE154918 sepsis plus septic shock (n = 39) versus healthy controls (n = 40); (3) GSE63042 sepsis death (n = 28) versus survivors from the uncomplicated, severe-sepsis and septic-shock groups (n = 78); and (4) GSE65682 28-day non-survivors (n = 114) versus survivors (n = 365). Figure 1E specifically examines genes shared by the two disease-versus-control screens, GSE63042 sepsis versus SIRS and GSE154918 sepsis/septic shock versus healthy. The GSE65682 pneumonia-sepsis versus non-infectious comparison was analyzed separately as an exploratory contrast. Gene sets for the four programmed cell death (PCD) programs (pyroptosis, necroptosis, ferroptosis, apoptosis) were curated from KEGG (hsa04621, hsa04217, hsa04216, hsa04210) and canonical literature (Tang et al., Cell Res 2019; Galluzzi et al., Cell Death Differ 2018; Stockwell et al., Cell 2017). Per-sample bulk program scores were computed as the mean of within-cohort z-scored member-gene expression.

**FIGURE 1:**
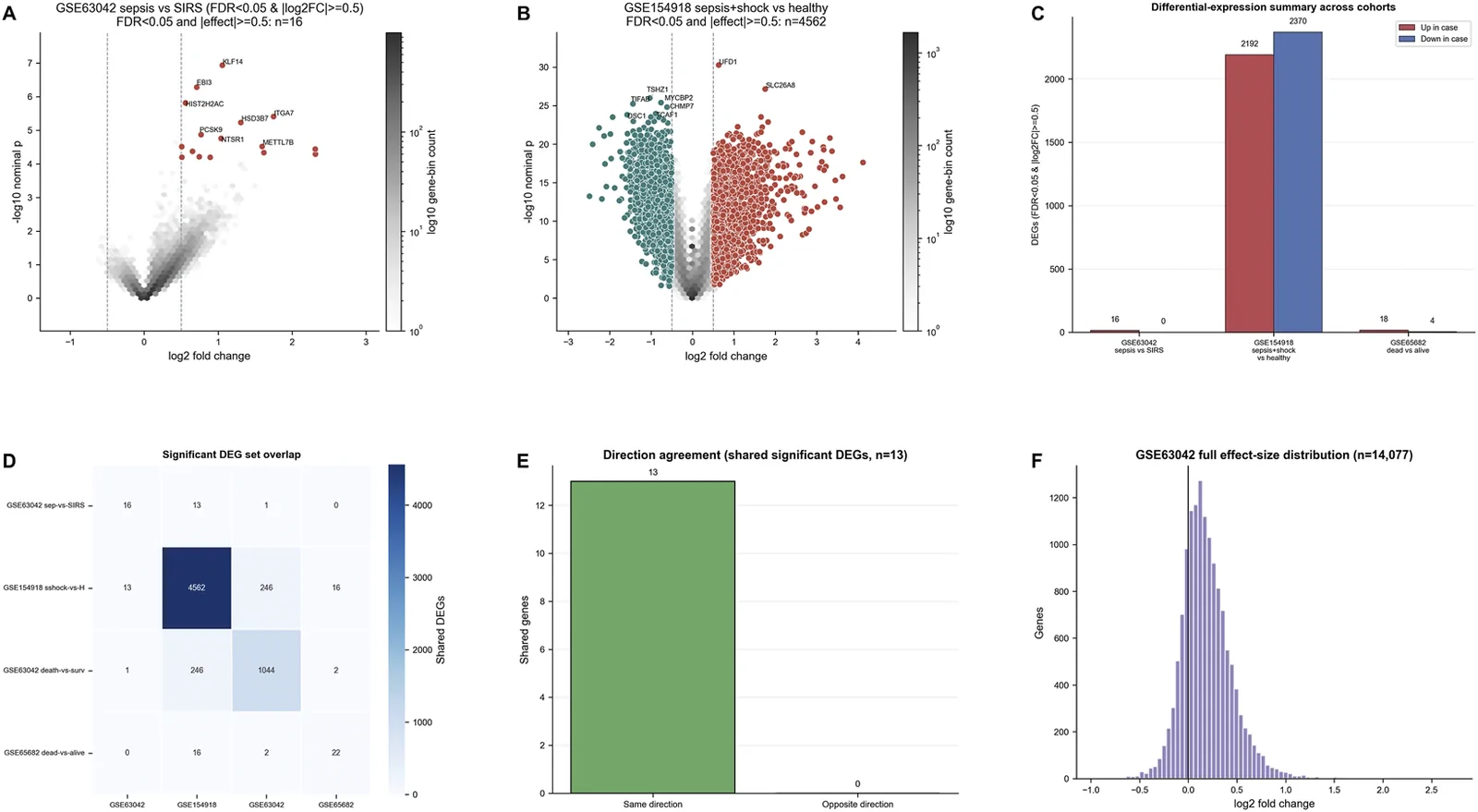
Differential expression across bulk cohorts with explicit contrast definitions. **(A)** Hexbin volcano of GSE63042 all sepsis groups (n = 106) versus SIRS (n = 23); y-axis -log10 nominal P; FDR<0.05 and |log2FC| ≥ 0.5 genes highlighted and top genes labeled. **(B)** Hexbin volcano of GSE154918 sepsis plus septic shock (n = 39) versus healthy controls (n = 40). **(C)** Counts of significant DEGs per contrast, including GSE63042 sepsis versus SIRS, GSE154918 sepsis/septic shock versus healthy, GSE63042 sepsis death (n = 28) versus survivors (n = 78), and GSE65682 28-day non-survivors (n = 114) versus survivors (n = 365). **(D)** Overlap matrix of these four explicitly labeled significant-DEG sets (diagonal = set size, off-diagonal = intersection); the largest displayed intersection is GSE63042 death-versus-survivor with GSE154918 sepsis/septic shock-versus-healthy (246 genes). **(E)** Direction agreement for the 13 DEGs shared specifically by the two disease-versus-control screens, GSE63042 sepsis-versus-SIRS and GSE154918 sepsis/septic shock-versus-healthy. **(F)** Histogram of the complete log2FC distribution of all tested genes in GSE63042 sepsis versus SIRS.

### Survival and outcome association

In GSE65682, 28-day mortality and time-to-event were analyzed for 479 sepsis patients with complete outcome data. For Kaplan-Meier visualization, patients were rank-ordered by each program score and divided into tertiles: low (n = 160), middle (n = 159), and high (n = 160). The Kaplan-Meier curves compared the low and high tertiles to maximize separation of the score distribution; the middle tertile was not plotted, which is why the displayed groups sum to 320 rather than 479. Log-rank tests compared the displayed low and high groups. Continuous core PCD-gene expression was evaluated in all 479 patients using univariate Cox proportional-hazards models (R lifelines). In GSE63042, the sepsis-death group (n = 28) was contrasted with survivors (n = 78) for core PCD gene effects.

### Single-cell processing, annotation and pseudobulk

All scRNA-seq libraries were read from the deposited count matrices. Cells with fewer than 200 detected genes or more than 15% mitochondrial reads were removed; genes detected in fewer than three cells were dropped. Counts were normalized to 10,000 per cell and log1p-transformed; the 2,000 most variable genes (Seurat v3 flavor) drove PCA (50 PCs), k-nearest-neighbor graph construction, UMAP and Leiden clustering (resolution 0.8, seed 42). Cell types were assigned by a DE-based marker-voting procedure against a curated lineage-marker panel; every cluster received a named cell type and a confidence record (Results/annotation_audit.csv). For cell-level PCD visualization, pyroptosis, necroptosis, ferroptosis and apoptosis scores were calculated from the same curated gene panels using Scanpy’s score_genes function (scanpy.tl.score_genes). These cell-level scores were treated descriptively; inferential comparisons were anchored at the independent library/patient level. Pseudobulk aggregates were formed by summing raw counts and normalizing to log2 CPM. Because GSE167363 sampled each patient twice (T0/T6), condition contrasts were aggregated to the patient level: 5 sepsis patients versus two healthy subjects, and two non-survivors versus three survivors. GSE151263 contributed one library per patient (3 ARDS versus 4 sepsis). Cell-type-resolved contrasts used patient-level sums within each cell type (minimum 10 cells per unit). All of these contrasts are exploratory given the small number of independent patients; P values should be interpreted descriptively.

### Pathway enrichment and GSEA

Differentially expressed genes were tested against GO Biological Process 2023 and KEGG 2021 Human with EnrichR (gseapy), separately for up- and downregulated genes. Gene set enrichment analysis (GSEA) used the preranked t-statistic from the GSE65682 dead-versus-alive contrast against KEGG pathway gene lists retrieved from the KEGG REST service and the curated PCD panels (1,000 permutations, seed 42).

### Cellular models and cultivation parameters

Two cell lines were used for the reported *in vitro* validation: RAW264.7 (murine macrophage-like cell line, ATCC TIB-71) and THP-1 (human monocytic cell line, ATCC TIB-202). Cells were maintained in RPMI-1640 medium (Gibco, catalog 11875–093) supplemented with 10% fetal bovine serum (Gibco, catalog 10270–106) and penicillin (100 U/mL)/streptomycin (100 μg/mL) (Gibco, catalog 15140–122). Cells were incubated at 37 °C with 5% CO_2_ in a humidified atmosphere. Cell identity was confirmed by short tandem repeat (STR) profiling, and *mycoplasma* absence was verified using the MycoAlert Detection System (Lonza, catalog LT07-318).

### Specimen collection and processing procedures

An LPS-stimulated inflammatory cell model was established by exposing cells to lipopolysaccharide (LPS; Sigma-Aldrich, catalog L2630) at 1 μg/mL for 24 h. Initial seeding density was 2 × 10^5^ cells per well in 6-well format. After stimulation, cells were washed twice with ice-cold PBS (0 °C–4 °C) and subjected to nucleic acid extraction. This model was used to assess transcriptional responses to LPS and was not intended to reproduce the full systemic complexity of clinical sepsis.

### Nucleic acid isolation and complementary DNA generation

Total RNA was extracted using TRIzol reagent (Invitrogen, catalog 15596026) per manufacturer’s instructions. RNA purity and concentration were assessed by NanoDrop 2000 spectrophotometry (Thermo Fisher Scientific), accepting A260/A280 ratios of 1.8–2.0. RNA integrity was verified by 1% agarose gel electrophoresis confirming intact 28S and 18 S rRNA bands at a 2:1 ratio. First-strand cDNA was synthesized from 1 μg RNA using the PrimeScript RT Kit with gDNA Eraser (Takara, catalog RR047 A) in 20 μL reactions at 37 °C for 15 min, with enzyme inactivation at 85 °C for 5 s.

### Amplification via quantitative real-time methodology

TB Green Premix Ex Taq II chemistry (Takara, catalog RR820 A) powered quantification on QuantStudio five instrumentation (Applied Biosystems). Individual 20 μL reactions comprised: 10 μL master mix (2× concentration), 0.8 μL each primer (10 μM), 0.4 μL ROX passive reference dye, 2 μL cDNA template (1:10 dilution), and 6 μL nuclease-free water. Cycling conditions: 95 °C pre-denaturation (30 s), followed by 40 cycles of 95 °C (5 s) and 60 °C (34 s). Melting curve analysis (60 °C–95 °C, 0.5 °C increments) confirmed amplicon specificity. All reactions were performed in triplicate with no-template controls.

Target gene primer sequences: CASP1 locus (168 bp): F 5′-TGA​GCC​AAG​AGT​CTG​TGC​TG-3′, R 5′-AGC​ACA​AAG​CTG​AAG​GAG​AC-3'. RIPK3 (175 bp): F 5′-CTG​CAG​AAG​CTG​AAG​ACC​ATC-3′, R 5′-GAG​CTC​GTC​CTT​GAT​GAG​CTT-3'. NLRP3 (152 bp): F 5′-TAC​CTG​ATC​ATC​GTG​TTC​AAC-3′, R 5′-CAG​GTC​ATG​CTC​TTG​AAC​TCA-3'. SIRT1 (164 bp): F 5′-AGC​TGA​AGG​TGA​CCA​AGA​TAC-3′, R 5′-CTC​CAG​GAT​GTT​GAG​CTT​GTA-3'. BCL2 (158 bp): F 5′-CAG​TAC​CTG​AAG​ACC​AAC​GAG-3′, R 5′-GTC​CTG​AAC​ATA​GGT​CTC​AAG-3'. HIF1A (171 bp): F 5′-TGG​ACA​AGC​TGT​TCG​AGA​AC-3′, R 5′-GAG​CTT​GTC​GAT​GAG​CTC​AAG-3'. Reference gene ACTB (β-actin, 150 bp): F 5′-CAT​GTA​CGT​TGC​TAT​CCA​GGC-3′, R 5′-CTC​CTT​AAT​GTC​ACG​CAC​GAT-3'. All primers were synthesized by Sangon Biotech (Shanghai, China) with HPLC purification.

### Statistical analysis

Statistical analyses for the transcriptomic datasets were implemented using the procedures described above, including Welch’s t-test, Benjamini–Hochberg correction, Kaplan-Meier/log-rank analysis, Cox proportional-hazards models, pseudobulk comparisons, enrichment analysis, and GSEA. Statistical significance was defined as specified for each analysis. The single-cell patient-level comparisons with small numbers of independent subjects were treated as exploratory. For RT-qPCR, triplicate PCR reactions were used to assess technical reproducibility; these technical replicates do not substitute for independent biological replication and the corresponding P values should be interpreted cautiously.

### Software and reproducibility

Analyses were implemented in Python 3.13 (scanpy 1.12.0, anndata 0.12.10, pandas 2.2.3, numpy 2.1.3, scipy 1.17.1, gseapy 1.2.1, lifelines 0.30.3, matplotlib 3.10.8) and R 4.4.3 (Bioconductor hgu219. db) for probe mapping.

## Results

### Cohort overview and the core PCD gene landscape in sepsis blood

The five cohorts contributed 19 scRNA-seq libraries (83,194 cells after QC) and 1,035 bulk samples (Figure 2A). In GSE63042, GSDMD, BCL2, GPX4 and SIRT1 showed their lowest group means in sepsis deaths, RIPK3 and MLKL were lowest in the SIRS group, S100A8/S100A9 peaked in severe sepsis, and HMGB1 and HIF1A were highest in the death group (Figure 2B). GSE154918 showed severity-graded shifts in core PCD gene expression along its healthy-to-septic-shock axis (Figure 2C). PCA of GSE63042 showed overlapping clouds with only partial separation between severity groups (Figure 2D). Scale audits confirmed log2 working scales for the microarray cohort (Figure 2E), and sample-sample correlations were uniformly high with no distinct group blocks (Figure 2F).

**FIGURE 2:**
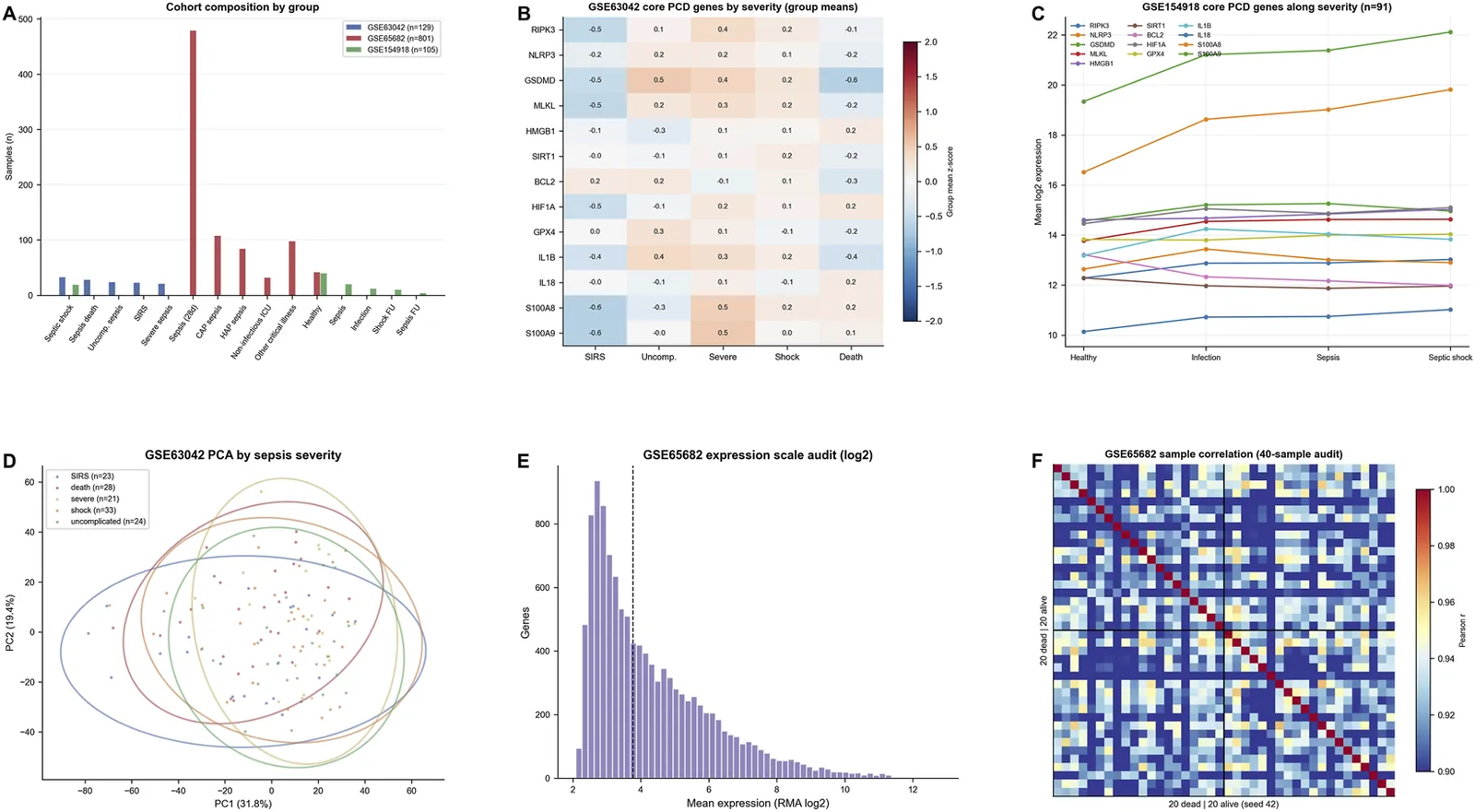
Cohort overview and PCD gene expression landscape. **(A)** Cohort composition with real sample counts per group for GSE63042 (n = 129), GSE65682 (n = 801) and GSE154918 (n = 105). **(B)** Group-mean row z-scored expression of the 13 core PCD genes quantified in GSE63042 across severity groups (SIRS n = 23, uncomplicated n = 24, severe n = 21, septic shock n = 33, sepsis death n = 28); CASP1 is not quantified in this cohort; values annotated per cell. **(C)** Mean log2 expression of core PCD genes along the GSE154918 severity gradient (cross-sectional n = 91; healthy 40, infection 12, sepsis 20, septic shock 19). **(D)** PCA (top 1,000 variable genes) of GSE63042 colored by severity with 95% confidence ellipses. **(E)** Histogram of gene means in GSE65682 confirming the RMA log2 working scale (median annotated). **(F)** Sample-sample Pearson correlation heatmap of a fixed 40-sample audit subset of GSE65682 (20 dead, 20 alive; seed 42), with group separator.

### The sepsis transcriptome signature is dominated by broad immune reprogramming

Sepsis versus SIRS in GSE63042 yielded 16 significant genes, all upregulated, whereas sepsis plus septic shock versus healthy controls in GSE154918 yielded 4,562 significant genes (2,192 up, 2,370 down; Figures 1A,B). The GSE65682 28-day non-survivor-versus-survivor contrast produced comparatively few significant genes (18 up, four down; Figure 1C). Figure 1D displays four explicitly labeled DEG sets: GSE63042 sepsis versus SIRS, GSE154918 sepsis/septic shock versus healthy, GSE63042 sepsis death versus survivors, and GSE65682 28-day non-survivors versus survivors. The largest intersection in this matrix was between GSE63042 death versus survivors and GSE154918 sepsis/septic shock versus healthy (246 genes). Figure 1E addresses a different question: it shows direction agreement for the 13 genes shared specifically by the two disease-versus-control screens (GSE63042 sepsis versus SIRS and GSE154918 sepsis/septic shock versus healthy). Because the central question concerns fatal outcome, the two death-versus-survival contrasts from GSE63042 and GSE65682 were treated as the principal outcome pair and were compared further at the core-gene level in Figures 3E,F. The complete effect distribution in GSE63042 sepsis versus SIRS was positively skewed (Figure 1F), indicating a broader tendency toward increased transcription even though only a small number of genes met the prespecified significance thresholds.

**FIGURE 3:**
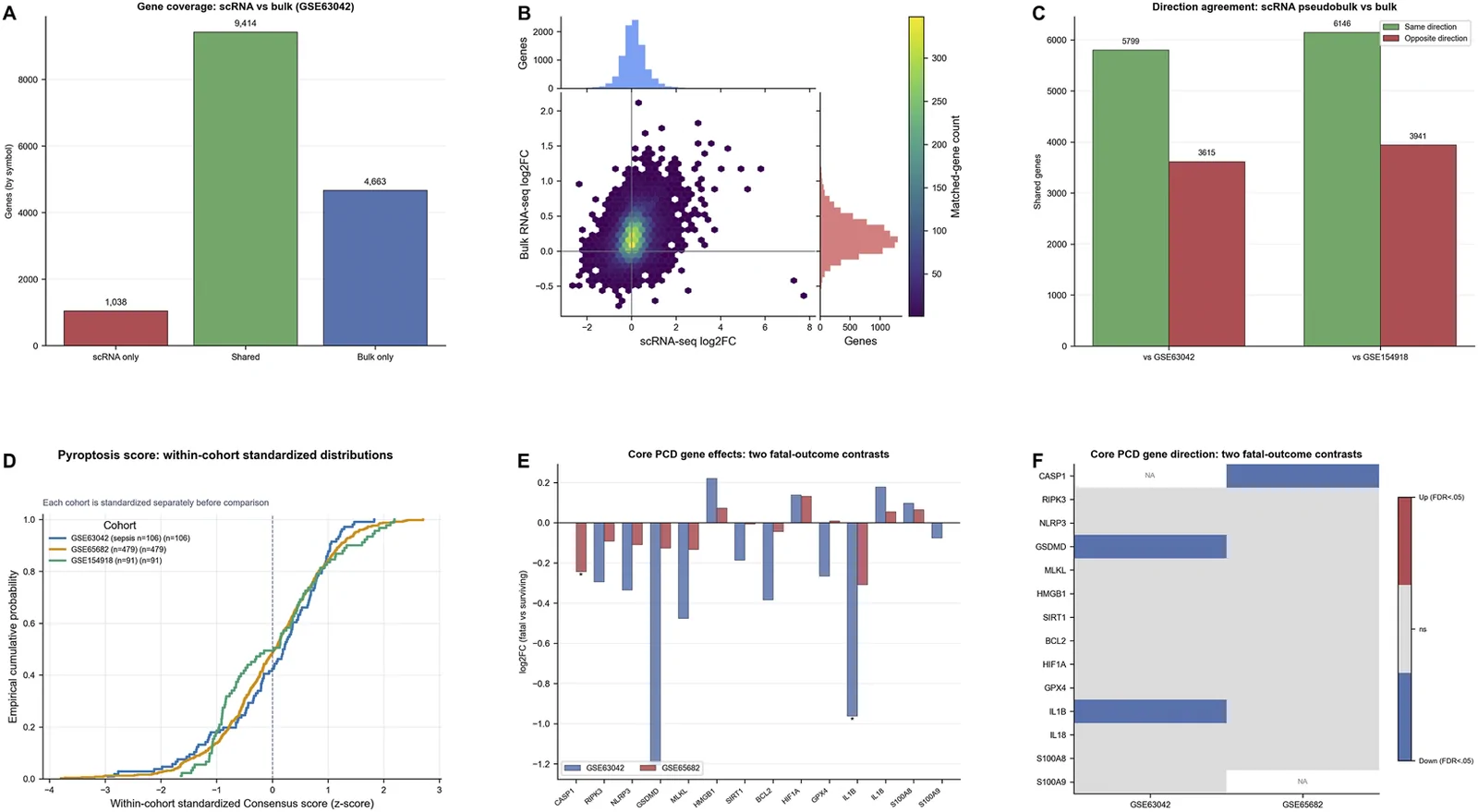
Cross-platform concordance and multi-cohort consistency. **(A)** Gene coverage: single-cell only, shared and bulk only (gene symbols). **(B)** Hexin concordance of patient-level pseudobulk and GSE63042 log2FC with marginal distributions. **(C)** Direction agreement of matched genes between patient-level pseudobulk and each bulk cohort. **(D)** Empirical CDFs of pyroptosis scores standardized within each cohort (GSE63042 sepsis n = 106, GSE65682 n = 479, GSE154918 n = 91). **(E)** Core PCD gene log2FC in the two outcome-focused fatal-sepsis contrasts: GSE63042 sepsis death-versus-survivor and GSE65682 28-day non-survivor-versus-survivor (grouped bars; * = FDR<0.05; no core gene is significant in both contrasts). **(F)** Direction matrix of core PCD genes across the same two fatal-outcome contrasts (red = up FDR<0.05, blue = down, gray = ns, NA = not quantified). Panels E–F emphasize that cross-cohort concordance is stronger at the program/pathway level than at the individual-gene level.

### Outcome-associated PCD programs: relative preservation of pyroptosis/necroptosis with survival and ferroptosis enrichment with death

The pyroptosis program score varied across the GSE63042 severity groups (Figure 4A). Within the GSE65682 sepsis cohort, 28-day non-survivors had lower pyroptosis, necroptosis and apoptosis scores but higher ferroptosis scores than survivors (Figure 4B). Kaplan-Meier analysis showed that patients in the high pyroptosis-score tertile had better 28-day survival than those in the low tertile (85.6% vs. 66.2%; log-rank P = 2.7e-05; Figure 4C); the same protective direction was observed for necroptosis (P = 0.00081) and apoptosis (P = 0.03), whereas high ferroptosis scores were associated with worse survival (P = 0.0034). In GSE63042, sepsis deaths showed downregulation of GSDMD (log2FC = −1.21, FDR = 3e-05) and IL1B (log2FC = −0.96, FDR = 0.01; Figure 4D), and Cox models in GSE65682 showed lower mortality hazards with higher CASP1 (HR = 0.57) and RIPK3 (HR = 0.43) expression (Figure 4E). By contrast, GSE154918 showed higher PCD-related program scores when infection/sepsis groups were compared with healthy controls (Figure 4F). These findings are not directionally contradictory because they address different contrasts: GSE154918 captures general disease-associated PCD transcriptional engagement relative to health, whereas GSE65682 asks whether relative program activity within established sepsis differs by outcome. Together, the datasets support a context-dependent model in which PCD programs can be induced during infection/sepsis overall, while relative preservation of pyroptosis/necroptosis within sepsis is associated with survival and ferroptosis enrichment is associated with fatal outcome.

**FIGURE 4:**
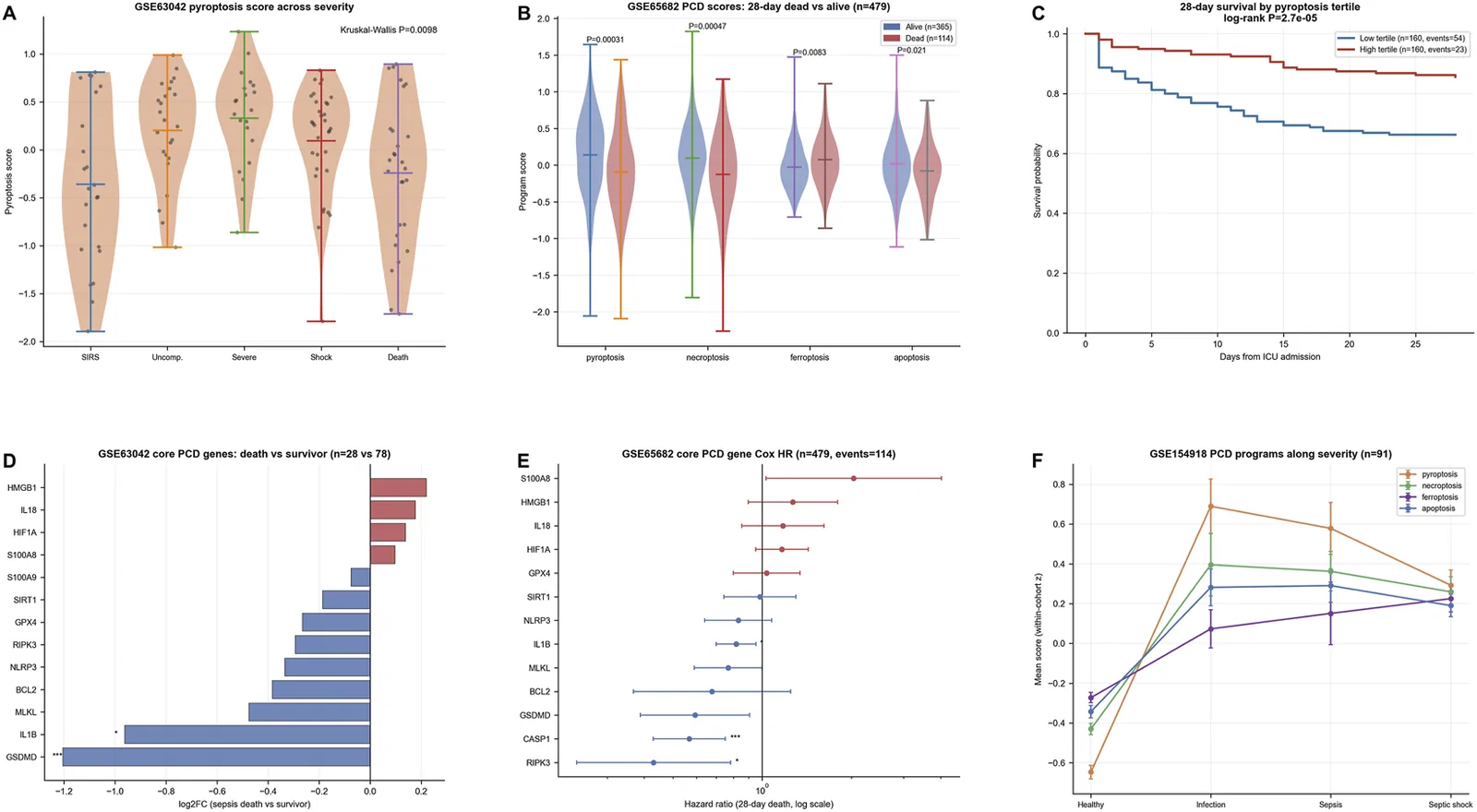
PCD program scores and 28-day outcome. **(A)** Pyroptosis program score across GSE63042 severity groups (per-patient; Kruskal–Wallis P). **(B)** Four PCD program scores in GSE65682 28-day non-survivors (n = 114) versus survivors (n = 365); Welch P per program. **(C)** Kaplan-Meier curves of GSE65682 by pyroptosis-score tertile. All 479 patients were divided into low (n = 160), middle (n = 159) and high (n = 160) tertiles; only low and high tertiles are displayed and compared by log-rank test. **(D)** Core PCD gene effects in GSE63042 sepsis death (n = 28) versus survivors (n = 78); stars: *FDR<0.05, **FDR<0.01, ***FDR<0.001. **(E)** Univariate Cox hazard ratios for core PCD genes in all GSE65682 sepsis patients with outcome data (n = 479, 114 events); x-axis log scale; S100A8 is nominal only (FDR = 0.106). **(F)** Mean ± SEM program scores along the GSE154918 severity gradient (cross-sectional n = 91). Panels A/F describe disease/severity context, whereas panels B–E provide the primary fatal-outcome evidence.

### Exploratory pneumonia-associated sepsis and ARDS analyses

The pneumonia/ARDS analyses were secondary and were not used as the primary evidence for the fatal-sepsis conclusion. In GSE65682, CAP and HAP sepsis patients had lower ferroptosis scores than non-infectious critically ill controls, but the overall difference was not statistically significant (Kruskal–Wallis P = 0.077; Figure 5A). The pneumonia-sepsis versus non-infectious contrast yielded 335 significant genes (134 up; Figure 5B), indicating broad transcriptional differences that were not specific to the fatal-outcome PCD signature. In GSE151263, sepsis-plus-ARDS and sepsis-only cells were extensively intermixed on UMAP, with differences in monocyte and lymphocyte composition (Figures 5C,D). Within pneumonia-associated sepsis in GSE65682, no core PCD gene differed between 28-day non-survivors and survivors at FDR<0.05 (minimum FDR = 0.186; Figure 5E), and cell-level pyroptosis scores were similar between sepsis and sepsis-plus-ARDS in GSE151263 (Figure 5F). These negative or weak findings are therefore presented as exploratory clinical context rather than support for the central mortality-associated transcriptional pattern.

**FIGURE 5:**
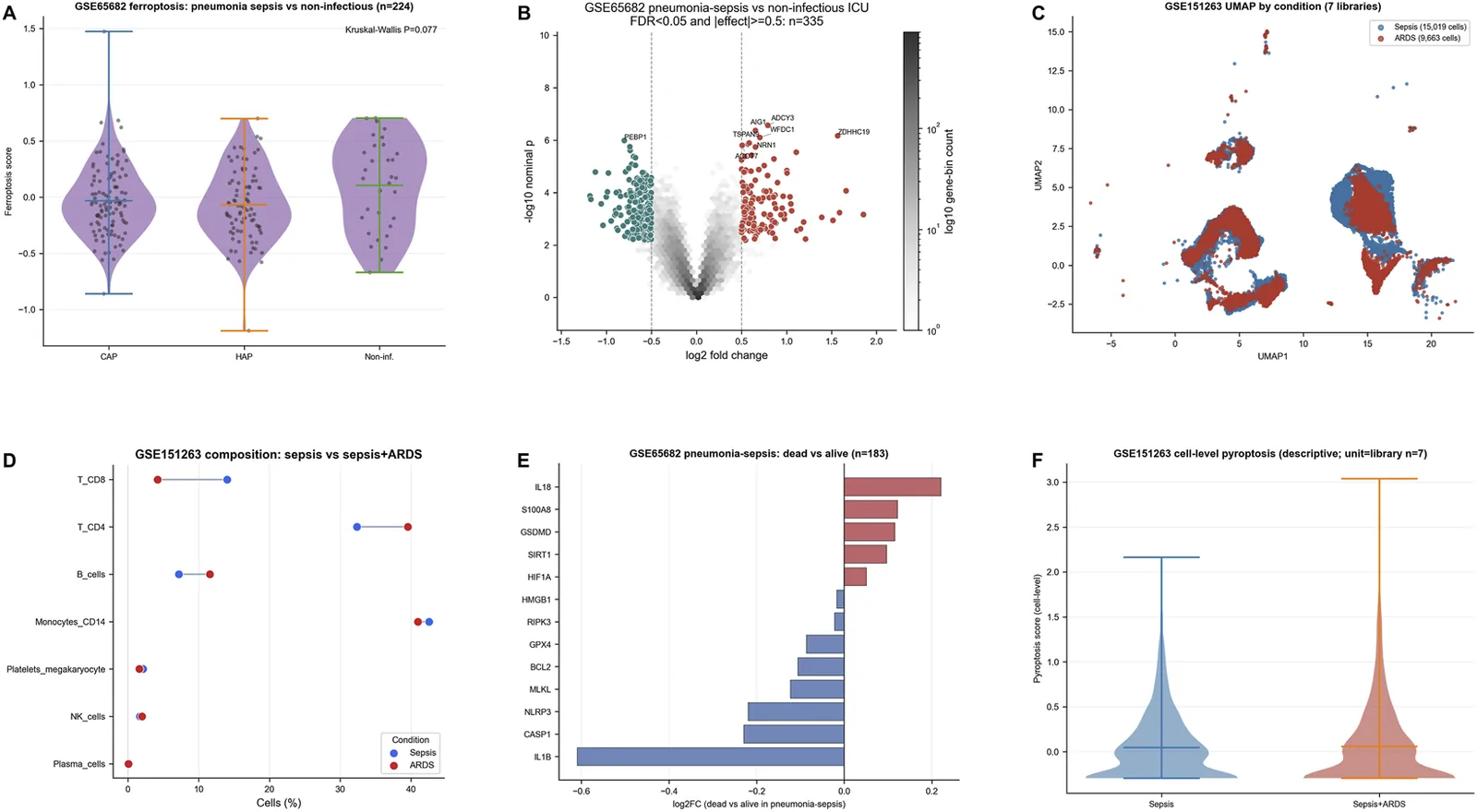
Exploratory pneumonia-associated sepsis and ARDS analyses. **(A)** Ferroptosis program score in GSE65682 CAP sepsis (n = 108), HAP sepsis (n = 84) and non-infectious critically ill controls (n = 32); Kruskal–Wallis P = 0.077. **(B)** Hexbin volcano of GSE65682 pneumonia sepsis (n = 192) versus non-infectious controls (n = 32). **(C)** UMAP of GSE151263 (7 libraries) colored by sepsis (n = 4) versus sepsis + ARDS (n = 3). **(D)** Cell-type composition dumbbell comparing the two GSE151263 conditions. **(E)** Core PCD gene effects between 28-day non-survivors and survivors within GSE65682 pneumonia sepsis; no gene passes FDR<0.05. **(F)** Cell-level pyroptosis score violins in GSE151263 (descriptive; statistical unit is the library, n = 7). These panels are secondary exploratory analyses and are not the principal evidence for the fatal-outcome PCD signature.

### Single-cell atlas, quality control and annotation

Integration of the two scRNA-seq cohorts yielded 83,194 cells from 19 libraries (Figure 6A), spanning healthy, sepsis and sepsis-plus-ARDS conditions (Figure 6B). Per-library retention after QC is shown in Figure 6C, and the UMI-versus-gene relationship after filtering is shown in Figure 6D. All clusters received DE-based cell-type annotations; most assignments had high confidence, and canonical lineage markers showed the expected cell-type distributions (Figures 6E,F). These QC and annotation results provided the basis for subsequent cell-type-resolved PCD scoring and pseudobulk comparisons rather than serving as outcome evidence by themselves.

**FIGURE 6:**
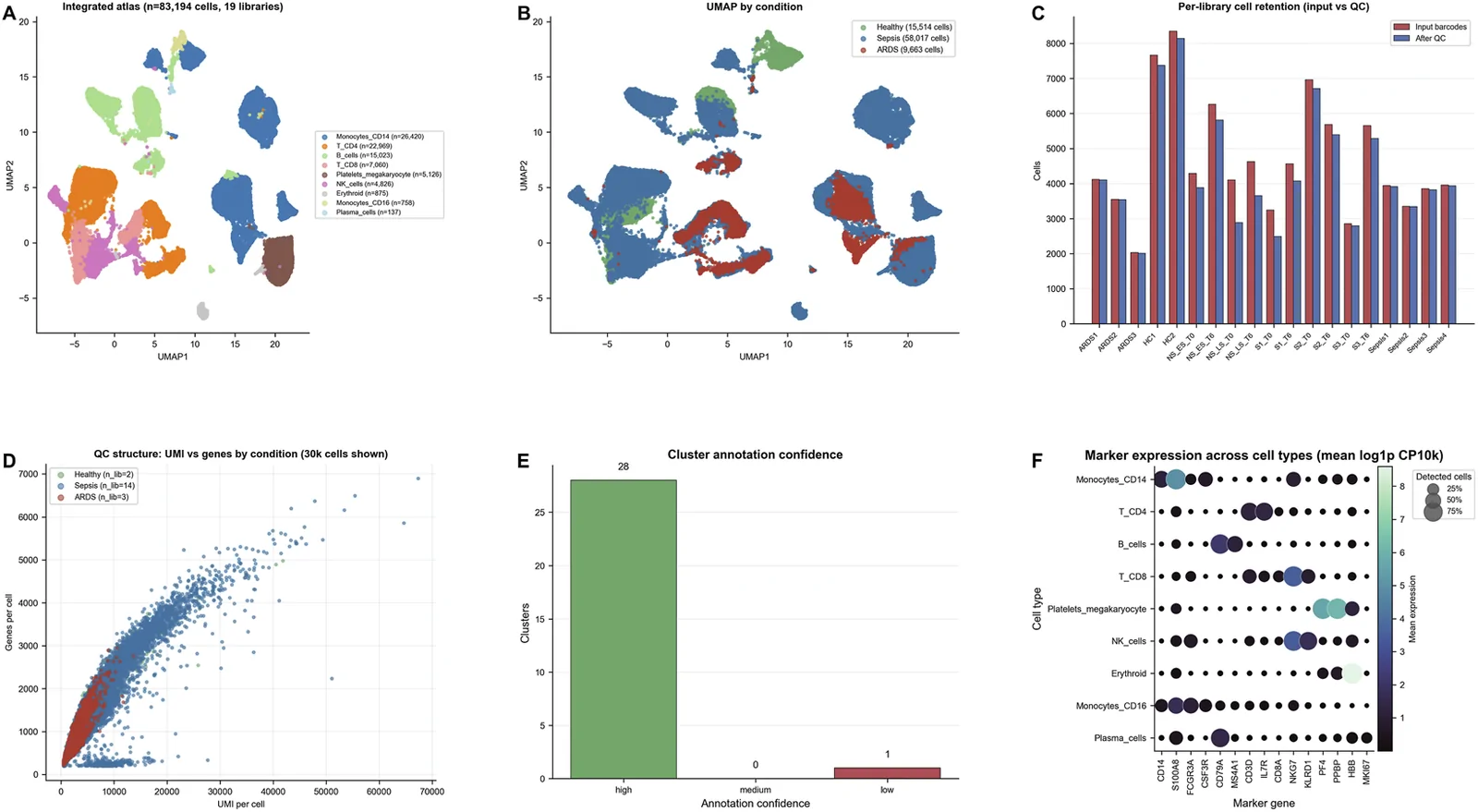
Single-cell atlas, QC and annotation. **(A)** Integrated UMAP colored by cell type (n cells annotated per type). **(B)** Integrated UMAP colored by condition (healthy, sepsis, sepsis + ARDS). **(C)** Per-library input versus post-QC cell counts (all 19 libraries). **(D)** QC scatter of UMI versus genes per cell colored by condition (30,000 cells shown, seed 5). **(E)** Annotation confidence counts across clusters (annotation_audit.csv). **(F)** Marker bubble matrix: bubble size = detection fraction, color = mean log-normalized expression per cell type.

### Cell-type-resolved PCD landscape and pseudobulk confirmation

Pyroptosis and ferroptosis scores were highest in CD14^+^ and CD16^+^ monocytes, with megakaryocytes additionally showing high ferroptosis scores, whereas T, B and NK lymphocytes generally had lower scores; necroptosis was most prominent in plasma cells and NK cells (Figures 7A–C). Core PCD genes showed a corresponding lineage pattern (Figure 7D), indicating that the bulk-blood PCD signal is not evenly distributed across immune-cell populations and is particularly prominent in monocyte compartments. In patient-level pseudobulk contrasts within GSE167363 (2 healthy vs. 5 sepsis patients; exploratory), sepsis was associated with GSDMD upregulation in monocyte, NK-cell and platelet units, together with lower NLRP3 and IL1B in sepsis monocytes (Figure 7E). Thus, individual genes within a pathway did not shift uniformly across all cell types. CASP1 and GSDMD were moderately co-expressed among cells expressing both genes (Spearman rho = 0.58, n = 3,974; Figure 7F), supporting coordinated transcription in a subset of cells without establishing pyroptotic execution.

**FIGURE 7:**
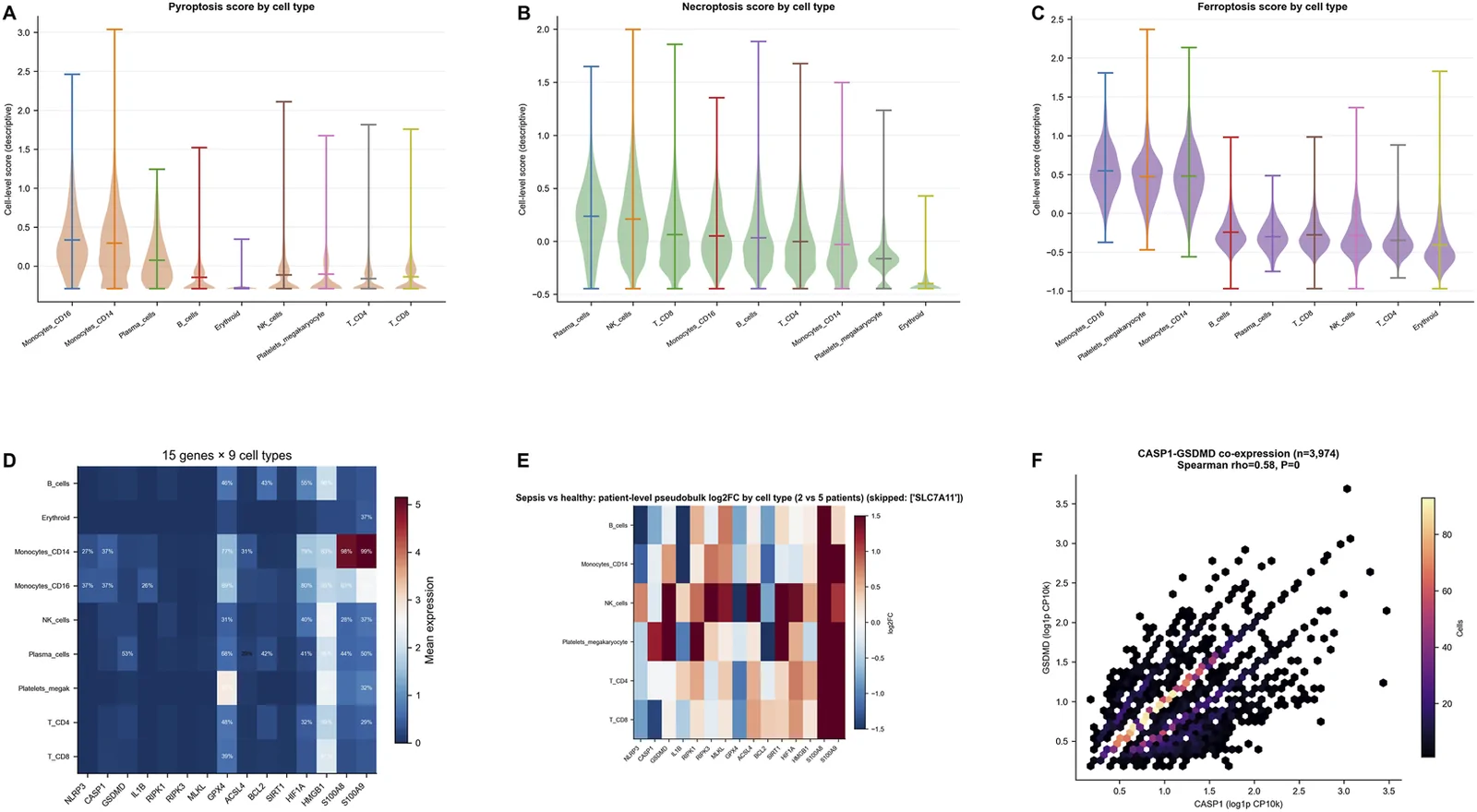
Cell-type-resolved PCD landscape. A-C. Cell-level pyroptosis **(A)**, necroptosis **(B)** and ferroptosis **(C)** scores by cell type, calculated from the curated PCD panels with scanpy. tl.score_genes (descriptive violin; max 2,500 cells per type shown; inference anchored at library/patient level). **(D)** Bubble plot of the 15 quantified core PCD genes by cell type (SLC7A11 not detected; size = detection fraction, color = mean log-normalized expression). **(E)** Patient-level pseudobulk log2FC heatmap of core PCD genes by cell type for sepsis versus healthy within GSE167363 (2 healthy vs. 5 sepsis patients; exploratory). **(F)** Hexbin co-expression of CASP1 and GSDMD across cells with both genes detected (log1p CP10k scale); Spearman rho and P annotated.

### Pathway and network architecture of sepsis PCD

Among genes upregulated in GSE65682 28-day non-survivors, KEGG terms were significantly enriched (minimum adjusted P = 6e-4; Figure 8A left), whereas genes downregulated in GSE63042 sepsis deaths did not yield a significant KEGG term (minimum adjusted P = 0.385; Figure 8A right). GSEA provided the clearest pathway-level outcome pattern: ferroptosis was enriched toward non-survivors (NES = 1.85, FDR = 0.00626), whereas pyroptosis (NES = −1.89, FDR = 0.001) and necroptosis (NES = −1.64, FDR = 0.00669) were enriched toward survivors (Figures 8B,C). The pathway-gene network showed that several outcome-associated terms shared member genes (Figure 8D), while the single-cell co-expression network was sparse, with only three pairwise correlations exceeding 0.35 and a dominant S100A8-S100A9 relationship (r = 0.94; Figure 8E). The dead-versus-alive pathway score heatmap reproduced the same directional split (Figure 8F). These analyses therefore support program-level, rather than uniformly gene-level, separation of fatal and non-fatal sepsis.

**FIGURE 8:**
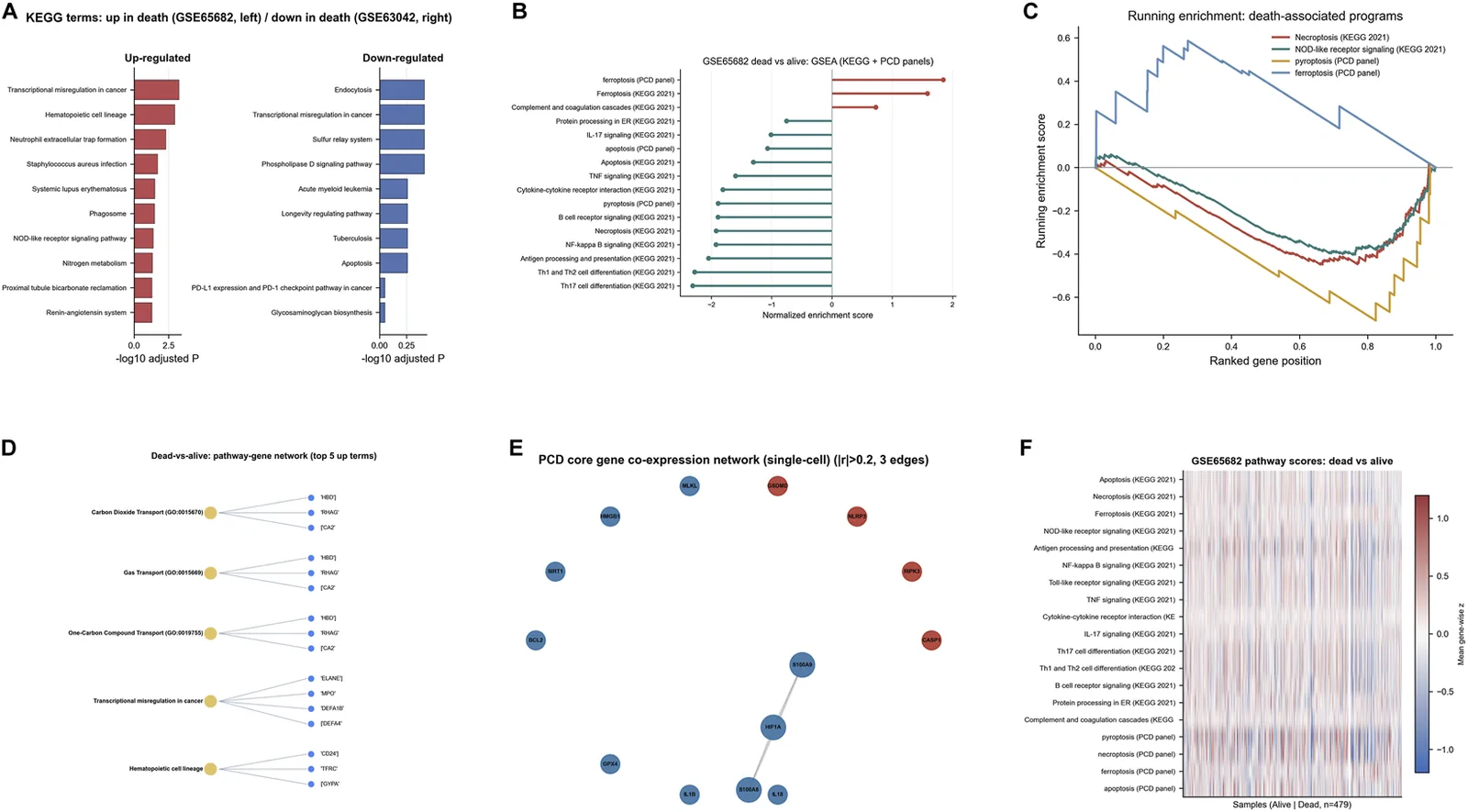
Pathway enrichment, GSEA and network architecture. **(A)** Top 10 KEGG terms: left = upregulated in GSE65682 28-day non-survivors (significant, min adjusted P = 6e-4); right = downregulated in GSE63042 sepsis deaths (descriptive, min adjusted P = 0.385). **(B)** Directional GSEA NES lollipop for KEGG 2021 and PCD panel gene sets in GSE65682 (ranked by t-statistic; 1,000 permutations). **(C)** Running enrichment curves for necroptosis, NOD-like receptor signaling, pyroptosis and ferroptosis gene sets. **(D)** Pathway-gene network of the top five terms upregulated in non-survivors (nodes = terms and up to six member genes each; edges = membership). **(E)** Single-cell co-expression network of the core PCD panel (Pearson on log-normalized expression; threshold 0.35, fallback heatmap at 0.2; only three edges exceed the threshold). **(F)** Pathway-level score heatmap across GSE65682 dead (n = 114) and alive (n = 365) samples with group separator (sample names omitted).

### Cross-platform concordance and multi-cohort consistency

Gene coverage overlapped substantially between the integrated single-cell atlas and the bulk cohorts (Figure 3A). Patient-level pseudobulk effects showed a modest but highly significant correlation with GSE63042 bulk effects (Spearman rho = 0.31, P = 5.9e-207, n = 9,414 aligned genes; Figure 3B), and matched-gene directions agreed above chance for both GSE63042 (61.6%) and GSE154918 (60.9%; Figure 3C). Standardized pyroptosis-score distributions were compared across all three bulk cohorts in Figure 3D to show cohort-level ordering after within-cohort scaling. Most importantly for the fatal-outcome question, the GSE63042 death-versus-survivor and GSE65682 non-survivor-versus-survivor contrasts were placed side-by-side in Figures 3E,F. No core PCD gene reached significance in both fatal-outcome cohorts, indicating that cross-cohort replication was stronger at the pathway/program level than at the level of individual genes. This distinction is important when interpreting the title and the proposed PCD signature.

### LPS-induced transcriptional responses of candidate PCD genes

RT-qPCR was used to test whether selected candidate PCD genes were transcriptionally responsive to acute LPS stimulation, not to reproduce the survivor-versus-non-survivor direction observed in clinical cohorts. CASP1 expression increased to 3.11 ± 0.37 in LPS-stimulated RAW264.7 macrophages and 2.68 ± 0.31 in THP-1 cells relative to the unstimulated control (P < 0.001). RIPK3 increased to 2.27 ± 0.34 and 1.95 ± 0.29, respectively (P < 0.01), and NLRP3 increased to 2.61 ± 0.38 and 2.19 ± 0.31 (P < 0.001). These results show that acute inflammatory stimulation can induce transcripts in pyroptosis- and necroptosis-related genes. They are therefore complementary to, rather than a directional replication of, the finding that relative pyroptosis/necroptosis program activity was lower in fatal clinical sepsis.

In contrast, SIRT1 expression decreased to 0.38 ± 0.07 in RAW264.7 and 0.45 ± 0.09 in THP-1 cells (P < 0.01), BCL2 to 0.42 ± 0.08 and 0.51 ± 0.10 (P < 0.05), and HIF1A to 0.47 ± 0.09 and 0.52 ± 0.11 (P < 0.05). The magnitude of change was generally greater in RAW264.7 than in THP-1 cells, which may reflect species- and lineage-related differences. Overall, the experiment confirms mRNA-level responsiveness of the six candidate genes to LPS. It does not demonstrate caspase-1 activation, RIPK3/MLKL signaling, gasdermin cleavage, membrane rupture or ferroptotic lipid peroxidation, and it should not be interpreted as functional validation of the fatal-sepsis pathway direction (Figure 9).

**FIGURE 9:**
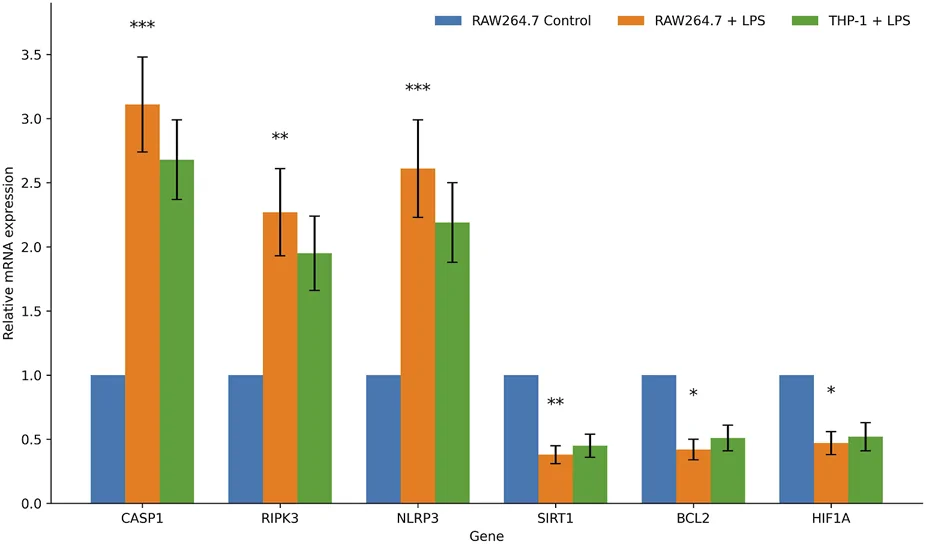
Real-time quantitative PCR analysis of mRNA expression levels of six PCD-related candidate genes in LPS-stimulated immune cell lines. The bar chart displays relative expression of CASP1, RIPK3, NLRP3, SIRT1, BCL2, and HIF1A in unstimulated RAW264.7 macrophages, LPS-stimulated RAW264.7 cells, and LPS-stimulated THP-1 cells. Values were normalized to the unstimulated RAW264.7 control (1.00). Error bars represent the variability of triplicate technical PCR reactions (n = 3 technical replicates). Statistical markers are shown as reported in the analysis; because independent biological replication was not performed, these P values should be interpreted cautiously. This experiment tests acute LPS-induced transcriptional responsiveness and is not a directional validation of the survivor-versus-non-survivor PCD pattern. It validates mRNA expression direction under LPS stimulation only and does not demonstrate functional activation of pyroptosis, necroptosis, or ferroptosis.

## Discussion

Our integrated analyses indicate that PCD-related transcriptional programs vary according to both disease context and clinical outcome. A broad rise in PCD-related scores from healthy controls to infection/sepsis in GSE154918 is compatible with activation of cell-stress and inflammatory transcriptional programs during disease development. The outcome comparison asks a different question: within GSE65682 sepsis patients, non-survivors showed relatively lower pyroptosis/necroptosis scores and higher ferroptosis scores than survivors, with concordant pathway-level survival associations. Thus, the manuscript does not infer that pyroptosis or necroptosis is globally suppressed throughout sepsis; rather, it identifies relative attenuation of these transcriptional programs in fatal outcome compared with surviving sepsis patients. Single-cell analyses further localized prominent pyroptosis- and ferroptosis-related transcriptional signals to monocyte populations. These findings remain transcriptional associations rather than direct evidence of pathway execution.

The RT-qPCR experiments address a different biological level and time context from the fatal-outcome cohort analyses. LPS stimulation increased CASP1, RIPK3 and NLRP3 mRNA and decreased SIRT1, BCL2 and HIF1A mRNA in RAW264.7 and THP-1 cells, demonstrating acute inflammatory transcriptional responsiveness. This does not contradict the lower pyroptosis/necroptosis program scores observed in clinical non-survivors, because an acute 24-h single-stimulus model is not a model of 28-day fatal outcome and does not capture immune exhaustion, treatment exposure, disease duration or multicellular systemic effects. Previous studies support roles for SIRT1 in immunometabolic and inflammatory regulation and for HIF-1α in macrophage metabolic responses (Liu et al., 2011; Vachharajani et al., 2016; Tannahill et al., 2013; Semba et al., 2016). Nevertheless, our data do not establish whether these expression changes are causes or consequences of cellular stress, nor do they demonstrate protein activation, cytokine release, membrane rupture or lipid peroxidation. The six genes should therefore be considered candidate transcriptional markers for further functional investigation rather than direct mechanistic confirmation of the fatal-sepsis signature.

Pneumonia-associated sepsis and ARDS analyses were included as secondary clinical context because pulmonary infection is a common source of sepsis (Hotchkiss et al., 2016; Delano and Ward, 2016; Matthay et al., 2019). These analyses did not provide strong outcome-specific support for the central PCD conclusion: the CAP/HAP ferroptosis-score comparison was not significant, no core PCD gene reached FDR<0.05 in the pneumonia non-survivor-versus-survivor comparison, and the GSE151263 ARDS analysis was limited by only seven independent libraries. Accordingly, Figure 5 should be interpreted as exploratory subgroup context rather than as a major line of evidence for the fatal-sepsis narrative. Proposed links among alveolar injury, DAMP release, inflammasome signaling, necroptosis and ferroptosis remain biological interpretations supported by prior literature (Matthay et al., 2019; Tang et al., 2012; Scaffidi et al., 2002; Huang et al., 2019; Li et al., 2020), not direct measurements in these datasets.

## Limitations

This study has several limitations. First, it is primarily a reanalysis of public bulk and single-cell transcriptomic datasets, and the cohorts differ in platform, clinical composition and comparison structure. Disease-versus-control contrasts and survivor-versus-non-survivor contrasts therefore should not be interpreted as interchangeable, and some apparent directional differences likely reflect the biological question being asked. Second, several single-cell comparisons include small numbers of independent patients and should be considered exploratory. Third, the *in vitro* component used an acute LPS-stimulated inflammatory cell model and does not reproduce the polymicrobial, multicellular, longitudinal or organ-specific environment of fatal clinical sepsis; its purpose was transcriptional responsiveness rather than directional replication of the mortality signature. Fourth, RT-qPCR measured mRNA expression only. No cleaved GSDMD, active caspase-1, phospho-MLKL, cytokine release, membrane-integrity or lipid-peroxidation assays were performed, so functional execution of pyroptosis, necroptosis or ferroptosis cannot be inferred. Fifth, no gain- or loss-of-function experiments were performed to establish causality. Sixth, cross-cohort consistency was stronger at the pathway/program level than at the individual core-gene level, as no core PCD gene was significant in both fatal-outcome contrasts. Seventh, RAW264.7 cells are murine whereas THP-1 cells are human, and species/lineage differences may contribute to the observed expression amplitudes. Future studies should include independent biological replication, primary human cells, pathway-specific protein and functional assays, genetic or pharmacological perturbation, and prospective clinical validation.

## Conclusion

In conclusion, this integrative analysis identifies context- and outcome-associated PCD transcriptional patterns across multiple sepsis cohorts. Relative to healthy controls, infection/sepsis can show broad engagement of several PCD-related programs; within established sepsis, however, fatal outcome was associated with relative suppression of pyroptosis- and necroptosis-related transcriptional programs and enrichment of ferroptosis-related signatures. The strongest cross-cohort consistency was observed at the program/pathway level rather than at individual-gene level. RT-qPCR in LPS-stimulated RAW264.7 and THP-1 cells showed that CASP1, RIPK3, NLRP3, SIRT1, BCL2 and HIF1A are transcriptionally responsive to acute inflammatory stimulation, but this experiment does not directionally or functionally validate the fatal-outcome signature. These genes and pathways remain candidate biomarkers or regulatory targets requiring protein-level, functional and prospective clinical validation before therapeutic or prognostic application.

## Data Availability

The datasets presented in this study can be found in online repositories. The names of the repository/repositories and accession number(s) can be found in the article/supplementary material.

## References

[B1] Delano M. J. Ward P. A. (2016). The immune system's role in sepsis progression, resolution, and long-term outcome. Immunol. Rev. 274 (1), 330–353. 10.1111/imr.12499 27782333 PMC5111634

[B2] Galluzzi L. Vitale I. Aaronson S. A. Abrams J. M. Adam D. Agostinis P. (2018). Molecular mechanisms of cell death: recommendations of the nomenclature committee on cell death 2018. Cell Death Differ. 25 (3), 486–541. 10.1038/s41418-017-0012-4 29362479 PMC5864239

[B3] He Y. Hara H. Nunez G. (2016). Mechanism and regulation of NLRP3 inflammasome activation. Trends Biochem. Sci. 41 (12), 1012–1021. 10.1016/j.tibs.2016.09.002 27669650 PMC5123939

[B4] Hotchkiss R. S. Moldawer L. L. Opal S. M. Reinhart K. Turnbull I. R. Vincent J. L. (2016). Sepsis and septic shock. Nat. Rev. Dis. Prim. 2, 16045. 10.1038/nrdp.2016.45 28117397 PMC5538252

[B5] Huang M. Cai S. Su J. (2019). The pathogenesis of sepsis and potential therapeutic targets. Int. J. Mol. Sci. 20 (21), 5376. 10.3390/ijms20215376 31671729 PMC6862039

[B6] Kang R. Zeng L. Zhu S. Shi Y. Liu J. Jia Y. (2018). Lipid peroxidation drives gasdermin D-Mediated pyroptosis in lethal polymicrobial sepsis. Cell Host Microbe 24 (1), 97–108. 10.1016/j.chom.2018.05.009 29937272 PMC6043361

[B7] Li J. Cao F. Yin H. L. Huang Z. J. Lin Z. T. Mao N. (2020). Ferroptosis: past, present and future. Cell Death Dis. 11 (2), 88. 10.1038/s41419-020-2298-2 32015325 PMC6997353

[B8] Linkermann A. Stockwell B. R. Krautwald S. Anders H. J. (2014). Regulated cell death and inflammation: an auto-amplification loop causes organ failure. Nat. Rev. Immunol. 14 (11), 759–767. 10.1038/nri3743 25324125

[B9] Liu T. F. Yoza B. K. El Gazzar M. Vachharajani V. T. McCall C. E. (2011). NAD+-Dependent SIRT1 deacetylase participates in epigenetic reprogramming during endotoxin tolerance. J. Biol. Chem. 286 (11), 9856–9864. 10.1074/jbc.M110.196790 21245135 PMC3058977

[B10] Matthay M. A. Zemans R. L. Zimmerman G. A. Arabi Y. M. Beitler J. R. Mercat A. (2019). Acute respiratory distress syndrome. Nat. Rev. Dis. Prim. 5 (1), 18. 10.1038/s41572-019-0069-0 30872586 PMC6709677

[B11] Pasparakis M. Vandenabeele P. (2015). Necroptosis and its role in inflammation. Nature 517 (7534), 311–320. 10.1038/nature14191 25592536

[B12] Qu M. Wang Y. Qiu Z. Zhu S. Guo K. Chen W. (2022). Necroptosis, pyroptosis, ferroptosis in sepsis and treatment. Shock 57 (6), 161–171. 10.1097/SHK.0000000000001936 35759299

[B13] Rudd K. E. Johnson S. C. Agesa K. M. Shackelford K. A. Tsoi D. Kievlan D. R. (2020). Global, regional, and national sepsis incidence and mortality, 1990-2017. Lancet 395 (10219), 200–211. 10.1016/S0140-6736(19)32989-7 31954465 PMC6970225

[B14] Scaffidi P. Misteli T. Bianchi M. E. (2002). Release of chromatin protein HMGB1 by necrotic cells triggers inflammation. Nature 418 (6894), 191–195. 10.1038/nature00858 12110890

[B15] Semba H. Takeda N. Isagawa T. Sugiura Y. Honda K. Wake M. (2016). HIF-1alpha-PDK1 axis-induced active glycolysis plays an essential role in macrophage migratory capacity. Nat. Commun. 7, 11635. 10.1038/ncomms11635 27189088 PMC4873978

[B16] Singer M. Deutschman C. S. Seymour C. W. Shankar-Hari M. Annane D. Bauer M. (2016). The third international consensus definitions for sepsis and septic shock (Sepsis-3). JAMA 315 (8), 801–810. 10.1001/jama.2016.0287 26903338 PMC4968574

[B17] Tang D. Kang R. Coyne C. B. Zeh H. J. Lotze M. T. (2012). PAMPs and DAMPs: signal 0s that spur autophagy and immunity. Immunol. Rev. 249 (1), 158–175. 10.1111/j.1600-065X.2012.01146.x 22889221 PMC3662247

[B18] Tannahill G. M. Curtis A. M. Adamik J. Palsson-McDermott E. M. McGettrick A. F. Goel G. (2013). Succinate is an inflammatory signal that induces IL-1beta through HIF-1alpha. Nature 496 (7444), 238–242. 10.1038/nature11986 23535595 PMC4031686

[B19] Vachharajani V. T. Liu T. Wang X. Hoth J. J. Yoza B. K. McCall C. E. (2016). Sirtuins link inflammation and metabolism. J. Immunol. Res. 2016, 8167273. 10.1155/2016/8167273 26904696 PMC4745579

[B20] van der Poll T. Shankar-Hari M. Wiersinga W. J. (2021). The immunology of sepsis. Immunity 54 (11), 2450–2464. 10.1016/j.immuni.2021.10.012 34758337

